# A novel marker for predicting malignancy in patients with thyroid nodules diagnosed as AUS on initial cytology: thyroid hormone sensitivity

**DOI:** 10.3389/fendo.2026.1825381

**Published:** 2026-05-12

**Authors:** Puren Gokbulut, Cagatay Emir Onder, Serife Mehlika Kuskonmaz, Huseyin Yagcı, Cavit Culha, Gonul Koc

**Affiliations:** Department of Endocrinology and Metabolic Diseases, Ankara Training and Research Hospital, Ankara, Türkiye

**Keywords:** atypia of undetermined significance (AUS), EU-TIRADS, malignancy risk stratification, thyroid hormone sensitivity indices, thyroid nodules

## Abstract

**Purpose:**

This study aimed to evaluate the predictive value of thyroid hormone sensitivity indices and hematological inflammatory markers, in addition to ultrasonographic features, for malignancy in thyroid nodules initially classified as atypia of undetermined significance (AUS).

**Methods:**

Data from 415 thyroid nodules initially diagnosed as Bethesda category III (AUS) on fine-needle aspiration biopsy (FNAB) were retrospectively analyzed. Nodules with benign histopathology or benign cytology on repeat FNAB were classified as the benign group, whereas those with malignant histopathology were classified as the malignant group. The groups were compared in terms of ultrasonographic features, baseline hematological parameters, and thyroid hormone sensitivity indices.

**Results:**

A total of 185 patients with thyroid nodules initially classified as Bethesda category III (AUS) were included, and 30.8% of surgically treated cases were malignant. A family history of thyroid cancer was more frequent in the malignant group (26.3% vs 10.9%, p=0.008). Malignant nodules were more likely to be solid, hypoechoic, and associated with microcalcifications, irregular margins, intranodular vascularity, and higher EU-TIRADS categories (all p<0.05). TSH and thyroid hormone sensitivity indices (TT4RI, TSHI, TFQI) were significantly higher in malignant nodules. In multivariable analysis, suspicious EU-TIRADS classification, family history of thyroid cancer, intranodular vascularity, PLR-to-PDW ratio, and thyroid hormone sensitivity indices remained independent predictors of malignancy. ROC analysis demonstrated moderate diagnostic performance for TSHI and TT4RI.

**Conclusion:**

Thyroid hormone sensitivity indices may serve as adjunctive markers for malignancy risk stratification in Bethesda category III thyroid nodules and may complement ultrasonographic assessment in clinical decision-making.

## Introduction

Differentiated Thyroid cancer (DTC) is the most common endocrine malignancy, with a rapidly increasing incidence in recent years. Thyroid nodules are highly prevalent and are incidentally detected in 20–67% of cases during ultrasonographic examinations (1). As thyroid nodules may pose a potential risk for malignancy, distinguishing between benign and malignant nodules is essential (2).

Fine-needle aspiration biopsy (FNAB) under ultrasonographic guidance is a globally utilized diagnostic tool for the evaluation of suspicious thyroid nodules. The Bethesda System for Reporting Thyroid Cytopathology is the standard diagnostic reporting system for FNAB, facilitating the accurate communication, interpretation, and sharing of cytopathological findings (3). The risk stratification approach based on cytological evaluation plays a critical role in the management of thyroid nodules by estimating the risk of malignancy and reducing unnecessary surgical interventions in benign nodules (1, 2, 4). Category III of the Bethesda System, currently termed ‘Atypia of Undetermined Significance (AUS)’ according to the third edition (2023), refers to cytological abnormalities that are not clearly benign or malignant (5). The reported frequency of Bethesda category III varies between 3% and 20.5%; however, repeat FNABs in these cases continue to yield indeterminate results in up to 65% of instances (6). Bethesda category III represents an indeterminate classification with an associated malignancy risk ranging from 6% to 30% (3). Ultrasonographic features including hypoechogenicity, irregular margins, microcalcifications, a taller-than-wide shape, and a solid composition are also considered significant predictors of malignancy (7). Several studies have suggested that biochemical markers such as TSH, thyroid hormones, anti-TPO antibodies, and inflammatory indices may contribute to malignancy detection and potentially enhance diagnostic accuracy (8–11). Thyroid hormone sensitivity can now be assessed centrally and peripherally using indices that have been developed. Central TH sensitization is related to the response of pituitary thyrotrope cells to circulating FT4 levels, which governs TSH secretion through the negative feedback mechanism of the hypothalamic-pituitary-thyroid (HPT) axis (12). Furthermore, parameters of thyroid hormone insensitivity, including TSH levels and TSH receptor expression, have been reported as potential biomarkers for evaluating aggressiveness and behavior in differentiated thyroid cancer (13). Inflammatory activation, a key factor in cancer development, has been shown to increase in malignant thyroid nodules, and the ratio of platelet-to-lymphocyte ratio (PLR) and platelet distribution width (PDW) values has been found to be a reliable inflammatory index in distinguishing cases with papillary thyroid cancer from benign nodules (14).

In this study, we aimed to investigate the diagnostic power of thyroid hormone sensitivity parameters and the platelet-to-lymphocyte ratio to platelet distribution width ratio (PLR-to-PDW ratio)PLR-PDW ratio, along with ultrasonographic features, in predicting malignancy in Bethesda category III (AUS/) thyroid nodules.

## Materials and methods

### Participants and data collection

**Figure 1:**
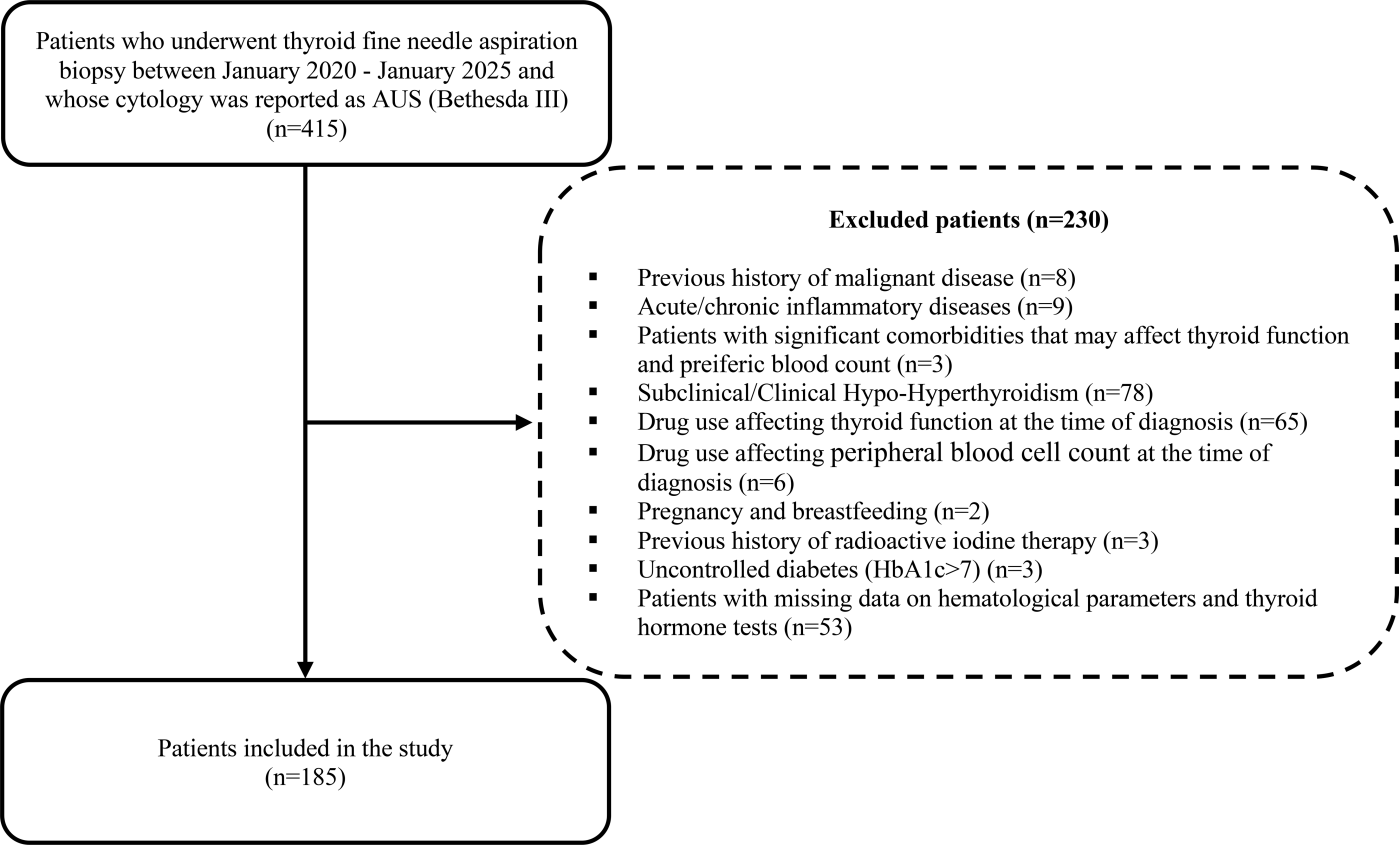
Flowchart for the selection of study population.

In our study, data from 415 thyroid nodules identified in patients aged 18 to 65 years—classified as Atypia of Undetermined Significance/(Bethesda category III) based on fine-needle aspiration (FNA) cytology performed at a tertiary medical care center within last five years—were retrospectively analyzed. Patients with a history of malignancy; those with coexisting conditions that could affect peripheral blood counts; individuals who are not euthyroid; pregnant or lactating women were excluded from the study. The flow diagram for the selection of the study population is presented in **Figure 1**. Demographic characteristics, clinical features, and thyroid ultrasonography (US) findings were reviewed from patient medical records. A family history of thyroid cancer was defined as the presence of thyroid cancer in at least one individual across two generations of the family.

### Ultrasonographic evaluation

Ultrasound reports performed by endocrinologists with at least 5 years of experience in thyroid ultrasonography in the endocrinology department were reviewed. Ultrasound (US) findings of the nodules—including size, topography, composition, echogenicity, shape, margin irregularity, presence of microcalcifications, and vascularity pattern—were recorded. The European Thyroid Imaging and Reporting Data System (EU-TIRADS) was used to categorize nodules based on their malignancy risk (15).

### Management evaluation

Following the cytological diagnosis of AUS, patients were classified according to their subsequent management: repeat fine-needle aspiration biopsy (FNAB), surgical intervention (total thyroidectomy or lobectomy), or follow-up with ultrasonography. For those who underwent repeat FNAB, cytology reports were reviewed, the updated Bethesda classification was recorded, and subsequent management decisions were analyzed. For patients who underwent surgery, histopathological reports were evaluated, and outcomes were categorized as benign or malignant. Patients with benign pathology as a result of histopathology and benign cytology as a result of RE-TFNAB were defined as the “Benign group”, and patients with malignant pathology as a result of histopathology were defined as the “Malignant group”.

### Laboratory evaluation

At the time of initial AUS diagnosis, baseline laboratory parameters were recorded. Blood samples were obtained as part of routine clinical evaluation following the initial AUS diagnosis, generally within approximately two weeks after FNAB in routine clinical practice. Patients with active infection or inflammatory conditions were excluded, and only euthyroid individuals not receiving thyroid-related medications were included in the analysis. Serum levels of thyroid-stimulating hormone (TSH) (mIU/L), free T4 (pmol/L), and anti-thyroid peroxidase antibodies (anti-TPO) (IU/mL) were retrieved from the hospital’s electronic database. Baseline complete blood count parameters—including neutrophil count (NE)(10³/µL), lymphocyte count (LY) (10³/µL), platelet count (PLT) (10³/µL), mean platelet volume (MPV) (fL), and platelet distribution width (PDW) (fL)—were also documented. Inflammatory indices were calculated as follows: platelet distribution width/platelet ratio (PPR) (fL/(10³/µL); platelet-to-lymphocyte ratio (PLR); neutrophil-to-lymphocyte ratio (NLR).

### Definition of TH sensitivity

The Thyrotrope Thyroxine Resistance Index (TT4RI), Thyroid Stimulating Hormone Index (TSHI) and Thyroid Feedback Quantile Based Index (TFQI), previously reported indices of central TH sensitivity, were used and calculated as follows:

- TT4RI= FT4 (pmol/L) × TSH (mIU/L)
- TSHI= Ln TSH (mIU/L) + 0.1345 × FT4 (pmol/L)
- TFQI= cdfFT4 - (1 - cdfTSH)

The TFQI is calculated with FT4 and TSH values according to an empirical cumulative distribution function and ranges from -1 to +1. An advantage of TFQI is that it does not produce extreme values and is more stable than TT4RI and TSHI. A negative TFQI indicates that the HPT axis is more sensitive to changes in FT4, while a positive TFQI indicates that the HPT axis is insensitive to FT4. A value of 0 means that the sensitivity of the HPT axis to FT4 changes is normal. Higher TSHI and TT4RI indices indicate a condition in which higher FT4 levels are inappropriately associated with normal or elevated TSH, i.e. low central sensitivity to thyroid hormone (16).

### Ethical approval

The study was approved by local Ethics Committee (date and number: November 11, 2024/E24-269). It was conducted in accordance with the Declaration of Helsinki.

### Statistical analysis

Statistical analyses were performed using IBM SPSS Statistics software (version 26). Continuous variables were tested for normality using the Kolmogorov–Smirnov test. Normally distributed variables are presented as mean ± standard deviation (SD), whereas non-normally distributed variables are expressed as median (minimum–maximum). Categorical variables are presented as numbers and percentages [n (%)]. Comparisons between patients with benign and malignant pathology were performed using the independent samples Student’s t-test for normally distributed continuous variables and the Mann–Whitney U test for non-normally distributed variables. Categorical variables were compared using the Chi-square (χ²) test or Fisher’s exact test, as appropriate. To identify potential predictors of malignancy, multiple logistic regression analyses were conducted. Variables with a p value < 0.20 on bivariate analysis or considered clinically relevant were eligible for inclusion in multivariable models. Results of logistic regression analyses are presented as odds ratio (OR) with 95% confidence intervals (CIs). To evaluate the diagnostic performance of peripheral blood cell indices and thyroid hormone sensitivity indices in distinguishing benign from malignant pathology, receiver operating characteristic (ROC) curve analysis was performed. The area under the ROC curve (AUC) with 95% confidence intervals was calculated. Optimal cut-off values were determined using the Youden index, and corresponding sensitivity and specificity values were reported. A two-sided p value < 0.05 was considered statistically significant.

### Sample size

Given the retrospective cross-sectional design of the study, no formal *a priori* sample size calculation was performed. Instead, all (n = 185) consecutive patients who met the predefined inclusion and exclusion criteria during the study period were enrolled. This consecutive-enrollment strategy was adopted to minimize selection bias and to ensure that the study sample reflected the full population of eligible patients encountered in routine clinical practice.

## Results

A total of 185 patients were included in the study. Their demographic, clinical, and ultrasonographic characteristics are presented in **Table 1**. Nodules with a largest diameter ≤1 cm accounted for 16.2% of all nodules. The management of patients initially diagnosed with AUS cytology is summarized in **Table 2**. Repeat fine-needle aspiration biopsy (FNAB) was performed in 89.2% of these patients. After repeat FNAB, cytology was reclassified as Bethesda II in 51.4% of patients, while 21.6% remained Bethesda III. Histopathological examination of patients who underwent surgery revealed thyroid malignancy in 30.8% of cases. Among patients who underwent surgery, papillary thyroid carcinoma was the most common malignancy, observed in 55 cases (29.7%). Within this group, the classic variant was the most frequent subtype (54.6%), followed by invasive encapsulated follicular variant (34.6%). Other variants, including oncocytic, tall cell, and infiltrative follicular variants, were less common. Follicular thyroid carcinoma was identified in 2 cases (1.1%), both of which were minimally invasive.

**Table 1:** Characteristics of the study population with AUS detected on initial cytology.

Demographic and clinical characteristics	Patients (n = 185)
Age (years)	52.56 ± 12.23
Gender [n (%)]	
Female	157 (84.9%)
Male	28 (15.1%)
BMI (kg/m^2^)	29.09 ± 3.35
Smoking [n (%)]^a^	60 (32.4%)
Diabetes Mellitus [n (%)]	37 (20%)
Chronic autoimmune thyroiditis [n (%)]	68 (36.8%)
Family history of thyroid cancer [n (%)]	29 (15.7%)
Nodule characteristics	
The largest diameter of nodule (cm)	1.97 (0.5 – 7.5)
Nodule localization [n (%)]	
Right lobe	85 (45.9%)
Left lobe	77 (41.6%)
Isthmus	23 (12.4%)
Settlement of the nodule in the thyroid gland [n (%)]	
Superior pol	27 (14.6%)
Middle section	90 (48.6%)
Inferior pol	44 (23.8%)
A whole lobe	1 (0.5%)
Isthmus	23 (12.4%)
Nodule composition [n (%)]	
Solid	122 (65.9%)
Mix (Solid and Cystic)	63 (34.1%)
Nodule echogenicity [n (%)]	
Hypoechoic	95 (51.4%)
Isoechoic	80 (43.2%)
Hyperechoic	10 (5.4%)
Presence of microcalcification [n (%)]	39 (21.1%)
Nodule margin [n (%)]	
Regular	160 (86.5%)
Irregular	25 (13.5%)
Nodule shape [n (%)]	
Taller-than-wide	10 (5.4%)
Wider-than-tall	175 (94.6%)
Presence of intranodular vascularity [n (%)]	26 (14.1%)
EU-TIRADS [n (%)]	
2	0 (0%)
3	71 (38.4%)
4	55 (29.7%)
5	59 (31.9%)

Normally distributed data are presented as mean ± SD, and non-normally distributed data are presented as median (min – max) in the table. Categorical variables are presented as n (%). ^a^Due to the lack of data on smoking status in all patients, the percentages calculated in the table were calculated among those with available data (n=157).

AUS, Atypia of undetermined significance, BMI, Body mass index, EU-TIRADS, European Thyroid Imaging and Reporting Data System.

**Table 2:** Management of patients after AUS detected on initial cytology.

Variable	Patients (n = 185)
Management after initial cytology AUS [n (%)]	
RE- FNAB	165 (89.2%)
Surgery	19 (10.3%)
Patient lost to follow-up	1 (0.5%)
Cytology after RE- FNAB [n (%)]	
Not available	20 (10.8%)
Bethesda I	17 (9.2%)
Bethesda II	95 (51.4%)
Bethesda III	40 (21.6%)
Bethesda IV	1 (0.5%)
Bethesda V	7 (3.8%)
Bethesda VI	5 (2.7%)
Management after RE- FNAB cytology [n (%)]	
Surgery	80 (48.5%)
Follow-up with USG	79 (47.9%)
Patient lost to follow-up	6 (3.6%)
Histopathology after surgery [n (%)]	
No surgery	86 (46.5%)
Benign pathologies	42 (22.7%)
Malignant pathologies	
Papillary thyroid carcinoma	55 (29.7%)
Invasive encapsulated follicular variant of papillary thyroid carcinoma	19 (34.6%)
Infiltrative follicular subtype	2 (3.6%)
Classic subtype	30 (54.6%)
Oncocytic subtype	3 (5.4%)
Tall cell subtype	1 (1.8%)
Follicular thyroid carcinoma	2 (1.1%)
Minimally invasive (capsular invasion only)	2 (100%)

Categorical variables are presented as n (%).

AUS, Atypia of undetermined significance, RE- FNAB: Repeat fine-needle aspiration biopsy, USG: Ultrasonography. The Bethesda 2023 classification was used in cytology. Bethesda I: Non-diagnostic, Bethesda II, Benign, Bethesda III, Atypia of undetermined significance (AUS), Bethesda IV, Follicular/Oncocytic neoplasia, Bethesda V, Suspicion of malignancy and Bethesda VI, Malignant.

**Table 3** compares the demographic, clinical, and ultrasonographic characteristics between benign and malignant nodules. No significant differences were observed between the groups regarding age, gender, BMI, smoking status, diabetes mellitus, chronic autoimmune thyroiditis, nodule size, localization, or nodule shape (p > 0.05). A family history of thyroid cancer was significantly more frequent in the malignant group (26.3% vs 10.9%, p = 0.008). Regarding ultrasonographic characteristics, malignant nodules were more frequently solid (75.4% vs 61.7%, p = 0.048), hypoechoic (73.7% vs 41.4%, p < 0.001), and associated with microcalcifications (42.1% vs 11.7%, p < 0.001), irregular margins (31.6% vs 5.5%, p < 0.001), and intranodular vascularity (26.3% vs 8.6%, p = 0.001). When stratified according to the EU-TIRADS classification, malignant nodules were predominantly categorized as EU-TIRADS 5, whereas benign nodules were most frequently classified as EU-TIRADS 3 (p < 0.001).

**Table 3:** Comparison of baseline and nodule characteristics of patients with benign and malignant pathology.

Variable	Benign group (n = 128) (cytology and histopathology)	Malignant group (n = 57) (histopathology)	p value
Demographic and clinical characteristics			
Age (years)	53.12 ± 12.80	51.29 ± 10.83	0.350
Gender [n (%)]			0.868
Female	109 (85.2%)	48 (84.2%)	
Male	19 (14.8%)	9 (15.8%)	
BMI (kg/m^2^)	29.40 ± 3.58	28.40 ± 2.66	0.060
Smoking [n (%)]^a^	42 (38.2%)	18 (38.3%)	0.989
Diabetes Mellitus [n (%)]	27 (21.1)	10 (17.5%)	0.577
Chronic autoimmune thyroiditis [n (%)]	49 (41.5%)	19 (38.8%)	0.742
Family history of thyroid cancer [n (%)]	14 (10.9%)	15 (26.3%)	0.008**
Nodule characteristics			
The largest diameter of nodule (cm)	1.67 (0.5-7.5)	1.6 (0.8-6.5)	0.600
Nodule localization [n (%)]			0.384
Right lobe	62 (48.4%)	23 (40.4%)	
Left lobe	49 (38.3%)	28 (49.1%)	
Isthmus	17 (13.3%)	6 (10.5%)	
Settlement of the nodule in the thyroid gland [n (%)]			0.371
Superior pol of lobe	18 (14.1%)	9 (15.8%)	
Middle section of lobe	59 (46.1%)	31 (54.4%)	
Inferior pol of lobe	34 (26.6%)	10 (17.5%)	
A whole lobe	0 (0%)	1 (1.8%)	
Isthmus	17 (13.3%)	6 (10.5%)	
Nodule composition [n (%)]			0.048*
Solid	79 (61.7%)	43 (75.4%)	
Mix (Solid and Cystic)	49 (38.3%)	14 (24.6%)	
Nodule echogenicity [n (%)]			<0.001***
Hypoechoic	53 (41.4%)	42 (73.7%)	
Isoechoic	65 (50.8%)	15 (26.3%)	
Hyperechoic	10 (7.8%)	0 (0%)	
Presence of microcalcification [n (%)]	15 (11.7%)	24 (42.1%)	<0.001***
Nodule margin [n (%)]			<0.001***
Regular	121 (94.5%)	39 (68.4%)	
Irregular	7 (5.5%)	18 (31.6%)	
Nodule shape [n (%)]			0.288
Taller-than-wide	5 (3.9%)	5 (8.8%)	
Wider-than-tall	123 (96.1%)	52 (91.2%)	
Presence of intranodular vascularity [n (%)]	11 (8.6%)	15 (26.3%)	0.001**
EU-TIRADS [n (%)]			<0.001***
2	0 (0%)	0 (0%)	
3	65 (50.8%)	6 (10.5%)	
4	39 (30.5%)	16 (28.1%)	
5	24 (18.8%)	35 (61.4%)	

Normally distributed data are presented as mean ± SD, and non-normally distributed data are presented as median (min-max) in the table. Categorical variables are presented as n (%). Student t test was used for parametric variables, Mann-Whitney U was used for nonparametric variables. Comparisons were made between categorical variables using Chi-square (χ2) or Fisher’s exact test. p<0.05 was accepted as statistically significant. Statistically significant p values are indicated in bold. *p < 0.05, **p < 0.01, ***p < 0.001. ^a^Due to the lack of data on smoking status in all patients, the percentages calculated in the table were calculated among those with available data (Benign group, n = 110; Malignant group, n = 47).

BMI, Body mass index, EU-TIRADS, European Thyroid Imaging and Reporting Data System.

**Table 4** presents a comparison of peripheral blood cell counts and thyroid-related indices between benign and malignant nodules. There were no statistically significant differences between malignant and benign nodules in terms of NE, LY, PLT, NLR, PLR, PDW or MPV (p > 0.05). However, a significant difference was observed in the PLR-to-PDW ratio between the two groups, with this ratio being significantly lower in patients with malignant nodules (p < 0.05). Among thyroid-related biomarkers and indices, TSH, TT4RI (Total T4 Resistance Index), and TSHI (TSH Index) levels were significantly higher in malignant nodules compared to benign ones (p < 0.05). TFQI values were significantly higher in malignant nodules (0.04 vs −0.09, p = 0.048).This indicates a decrease in thyroid hormone sensitivity in the malignant group.

**Table 4:** Comparison of peripheral blood cell count and thyroid-related indexes between patients with benign and malignant pathology.

	Benign group (n = 128) (cytology and histopathology)	Malignant group (n = 57) (histopathology)	p value
Peripheral blood cell count and related indexes			
Neutrophil count (10^3^/mm^3^)	4.34 ± 1.65	4.79 ± 1.82	0.125
Lymphocyte count (10^3^/mm^3^)	2.35 ± 0.81	2.43 ± 0.8	0.553
Platelet count (10^3^/mm^3^)	291.41 ± 77.09	286.22 ± 67.31	0.787
NLR	2.03 ± 1.03	2.06 ± 0.83	0.484
PLR	136.21 ± 57.34	124.34 ± 35.07	0.236
PDW(fL)	12.82 ± 2.46	13.39 ± 2.23	0.084
PLR-to-PDW ratio	10.95 ± 5.17	9.53 ± 3.33	0.045*
MPV (fL)	10.48 ± 1.25	10.79 ± 1.46	0.058
Thyroid-related biomarkers and indexes			
TPO Ab (IU/mL)	11.35 (6 – 900)	12.4 (6 – 441)	0.541
Tg Ab (IU/mL)	16.1 (3 – 4030)	14.9 (1 – 561)	0.369
FT4 (pmol/L)	15.06 (11.97 – 21.88)	14.67 (11.97 – 21.88)	0.677
TSH (mIU/L)	1.29 (0.49 – 4.33)	1.79 (0.50 – 4.33)	0.003**
TFQI	-0.09 (-0.8 – 0.83)	0.04 (-0.83 – 0.89)	0.048*
TT4RI	18.75 (7.25 – 76.92)	25.01 (7.06 – 78.20)	0.003**
TSHI	2.25 (1.28 – 3.89)	2.33 (1.08 – 3.96)	0.004**

Normally distributed data are presented as mean ± SD, and non-normally distributed data are presented as median (min-max) in the table. Student t test was used for parametric variables, Mann-Whitney U was used for nonparametric variables. p<0.05 was accepted as statistically significant. Statistically significant p values are indicated in bold. *p < 0.05, **p < 0.01.

MPV, Mean platelet volume, NLR, Neutrophil-to-lymphocyte ratio, PDW, Platelet distribution width, PLR platelet-to-lymphocyte ratio, PLR-to-PDW ratio, PLR/PDW, Tg Ab, Thyroglobulin Antibody, TPO Ab, Thyroid Peroxidase Antibody, TSH, Thyroid-stimulating hormone, TFQI, Thyroid Feedback Quantile Based Index = cdfsT4-(1-cdfTSH), TSHI, Thyroid Stimulating Hormone index = InTSH+0.1345xFT4, TT4RI, Thyrotropic Thyroxine Resistance Index = FT4xTSH.

### Bivariate and multivariate analysis

To identify candidate predictors for multivariable modeling, bivariate analyses were first performed, and variables with clinical relevance or a p value <0.20 in bivariate analysis were considered for multivariable modeling. On bivariate analysis; Family history of thyroid cancer, nodule composition, echogenicity, the presence of microcalcifications, nodule margin, intranodular vascularity, EU-TIRADS classification, neutrophil count, platelet distribution width (PDW), mean platelet volume (MPV), PLR-to-PDW ratio, TSH, TT4RI, and TSHI were each significantly associated with malignant histopathology. Because several ultrasonographic features (such as nodule composition, echogenicity, microcalcification, and margin irregularity) are components of established risk stratification systems, individual sonographic characteristics were not entered separately into the multivariable model in order to avoid overfitting and model instability. Instead, the EU-TIRADS classification was used as a composite ultrasonographic risk variable. Similarly, rather than entering individual peripheral blood cell count and thyroid hormone parameters separately, derived indices calculated from these parameters were incorporated into the regression models. Due to the strong correlation between the thyroid hormone sensitivity indices TSHI (≥ 2.6 vs < 2.6) and TT4RI (≥ 27.67 vs < 27.67) (r = 0.89, p < 0.001), simultaneous inclusion of both variables in a single model was avoided to prevent multicollinearity-related instability in regression coefficients. Therefore, two separate multivariable logistic regression models were constructed: one incorporating TSHI (Model 1) and the other incorporating TT4RI (Model 2), with EU-TIRADS classification, family history of thyroid cancer, intranodular vascularity, and PLR-to-PDW ratio included in both. Potential confounders — age, body mass index (BMI), and diabetes mellitus status — were introduced individually and then simultaneously in both models to assess their effect on the primary associations. These variables were selected as potential confounders based on prior clinical knowledge and literature.

In both models, suspicious EU-TIRADS classification (EU-TIRADS 4–5) emerged as the strongest independent predictor of malignancy. Intranodular vascularity and a family history of thyroid cancer were also identified as significant predictors. Among the thyroid hormone sensitivity indices, TSHI ≥ 2.6 (Model 1) and TT4RI ≥ 27.67 (Model 2) were each independently associated with malignancy and demonstrated comparable effect sizes. Although the PLR-to-PDW ratio did not show meaningful discriminative performance in ROC analysis, it remained a statistically significant independent predictor in both multivariable models, with higher values inversely associated with the risk of malignancy. None of the potential confounding variables (age, body mass index, or diabetes mellitus status) reached statistical significance in any model specification. Moreover, their inclusion—either individually or simultaneously—did not materially alter the primary associations, supporting the robustness of the observed relationships. Detailed results of the multivariable logistic regression analyses are presented in **Table 5**.

**Table 5:** Multiple logistic regression analysis to evaluate independent predictors of thyroid malignancy in nodules with AUS cytology.

Independent variables	Model 1 (base)	Adjusted age	Adjusted BMI	Adjusted Dm status	Adjusted age + BMI + Dm status
Family history of thyroid cancer (Yes vs No)	3.965** (1.433 – 10.968)	3.864* (1.390 – 10.738)	3.811* (1.362 – 10.663)	3.961** (1.427 – 10.998)	3.790* (1.350 – 10.637)
EU-TIRADS (Suspicious vs non-suspicious)^a^	10.765*** (3.888 – 29.805)	10.701*** (3.869 – 29.603)	11.479*** (4.047 – 32.555)	10.781*** (3.892 – 29.863)	11.439*** (4.031 – 32.464)
Intranodular vascularity (Yes vs No)	3.935** (1.402 – 11.050)	3.794* (1.337 – 10.767)	3.831* (1.378 – 10.652)	3.927** (1.397 – 11.037)	3.797* (1.354 – 10.650)
PLR-to-PDW ratio	0.888* (0.801 – 0.986)	0.885* (0.797 – 0.983)	0.895* (0.807 – 0.992)	0.886* (0.798 – 0.984)	0.894* (0.805 – 0.993)
TSHI (≥ 2.6 vs < 2.6)	3.404** (1.570 – 7.380)	3.452** (1.585 – 7.519)	3.643** (1.650 – 8.044)	3.339** (1.534 – 7.266)	3.653** (1.640 – 8.134)
Confounding					
Age (years)	–	0.990 (0.958 – 1.023)	–	–	0.998 (0.964 – 1.033)
BMI (kg/m^2^)	–	–	0.893 (0.787 – 1.014)	–	0.895 (0.783 – 1.024)
Dm status	–	–	–	1.274 (0.497 – 3.265)	0.992 (0.364 – 2.703)
Independent Variables	Model 2 (Base)	Adjusted Age	Adjusted BMI	Adjusted Dm status	Adjusted Age + BMI + Dm status
Family history of thyroid cancer (Yes vs No)	3.797* (1.355 – 10.635)	3.652* (1.295 – 10.303)	3.686* (1.297 – 10.471)	3.798* (1.351 – 10.675)	3.624* (1.270 – 10.339)
EU-TIRADS (Suspicious vs non-suspicious)^a^	11.896*** (4.231 – 33.444)	11.838*** (4.213 – 33.264)	12.752*** (4.422 – 36.774)	11.886*** (4.228 – 33.414)	12.668*** (4.393 – 36.528)
Intranodular vascularity (Yes vs No)	4.365** (1.540 – 12.374)	4.141** (1.443 – 11.883)	4.197** (1.501 – 11.740)	4.347** (1.532 – 12.332)	4.104** (1.451 – 11.604)
PLR-to-PDW ratio	0.885* (0.796 – 0.983)	0.880* (0.791 – 0.979)	0.891* (0.802 – 0.988)	0.883* (0.794 – 0.981)	0.889* (0.799 – 0.988)
TT4RI (≥ 27.67 vs < 27.67)	4.148** (1.857 – 9.267)	4.307*** (1.907 – 9.728)	4.474*** (1.962 – 10.201)	4.069** (1.813 – 9.132)	4.561*** (1.972 – 10.551)
Confounding					
Age (years)	–	0.987 (0.955 – 1.020)	–	–	0.994 (0.960 – 1.029)
BMI (kg/m^2^)	–	–	0.890 (0.783 – 1.012)	–	0.894 (0.781 – 1.024)
Dm status	–	–	–	0.813 (0.313 – 2.113)	1.072 (0.386 – 2.974)

Data are presented as OR (Odds ratio) (95% CI (Confidence intervals)). Bold OR indicates statistical significance (*p < 0.05, **p < 0.01, ***p < 0.001).

BMI, Body mass index, Dm, Diabetes mellitus, EU-TIRADS, European Thyroid Imaging and Reporting Data System, PLR-to-PDW ratio, PLR/PDW, TSHI, Thyroid Stimulating Hormone index = InTSH+0.1345xFT4, TT4RI, Thyrotropic Thyroxine Resistance Index = FT4xTSH. ^a^:The suspicious category represents EU-TIRADS 4 and 5, and the non-suspicious category represents EU-TIRADS 3.

### ROC curves of peripheral blood cell count–derived and thyroid-related indices

**Figure 2:**
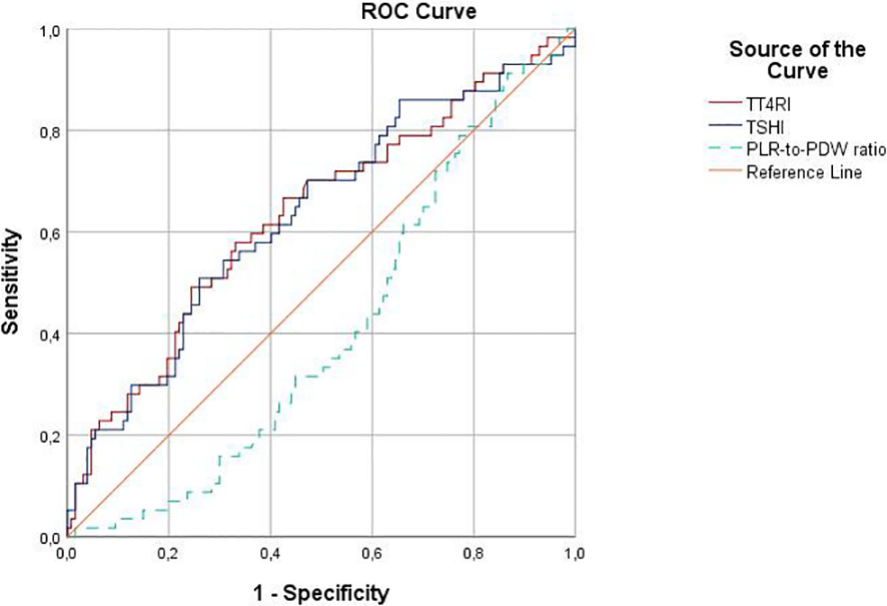
Receiver operating characteristic (ROC) curves for distinguishing malignant from benign histopathology in patients with atypia of undetermined significance (AUS) on initial cytology (n = 185), based on peripheral blood cell indices and thyroid hormone sensitivity indices.

In ROC analysis, TT4RI and TSHI showed modest but statistically significant discriminatory ability for predicting malignancy. A TT4RI cut-off value of ≥ 27.67 yielded 49.1% sensitivity and 75.0% specificity (AUC, 0.636**;** 95% CI, 0.547–0.725**;** *p* = 0.003), whereas a TSHI cut-off value of ≥ 2.6 yielded 50.8% sensitivity and 74.2% specificity (AUC, 0.634**;** 95% CI, 0.545–0.723; *p* = 0.004) for the prediction of malignancy. Although the original ROC analysis for the PLR-to-PDW ratio yielded an AUC below 0.50, indicating inverse directional discrimination, lower values were associated with malignancy. Therefore, for interpretive clarity, the ROC curve was reinterpreted after reversing the classification direction, and the corresponding AUC was presented as 1 − original AUC, yielding an AUC of 0.593 (95% CI, 0.509–0.676**;** *p* = 0.045). Notably, the PLR-to-PDW ratio remained significantly associated with malignancy in the multivariable logistic regression models and was therefore retained as a potential independent predictor (**Figure 2**).

### Exploratory combined analysis of ultrasonographic and thyroid hormone sensitivity indices

To further evaluate whether the diagnostic performance of thyroid hormone sensitivity indices could be enhanced by integration with ultrasonographic risk stratification, an exploratory combined analysis was performed using suspicious EU-TIRADS categories (EU-TIRADS 4–5) together with elevated TSHI or TT4RI. When test positivity was defined as the concurrent presence of suspicious EU-TIRADS and TSHI ≥ 2.6, the sensitivity and specificity for malignancy were 43.9% and 86.7%, respectively. Similarly, the combination of suspicious EU-TIRADS and TT4RI ≥ 27.67 yielded a sensitivity of 42.1% and a specificity of 89.1%. Overall, these findings indicate that combining suspicious ultrasonographic features with thyroid hormone sensitivity indices improves specificity, but does not enhance sensitivity.

## Discussion

In this study, we evaluated the predictive value of thyroid hormone sensitivity indices and hematological inflammatory parameters in addition to ultrasonographic features for malignancy in thyroid nodules initially classified as Bethesda category III (AUS). Our findings demonstrated that suspicious EU-TIRADS classification, intranodular vascularity, and a family history of thyroid cancer were strong independent predictors of malignancy. In addition, indices reflecting decreased central thyroid hormone sensitivity (TSHI and TT4RI) were independently associated with malignant pathology. These findings suggest that thyroid hormone sensitivity indices may provide complementary information to ultrasonographic risk stratification in AUS nodules.

The management of Bethesda category III nodules remains challenging, and current approaches increasingly rely on individualized risk stratification combining cytological, ultrasonographic, and clinical parameters (8, 17).

In recent years, molecular testing methods such as the Afirma Genomic Sequencing Classifier and ThyroSeq have been increasingly incorporated into the diagnostic algorithm for indeterminate thyroid nodules, particularly those classified as Bethesda category III (AUS). These approaches have demonstrated high diagnostic performance, especially in terms of negative predictive value, and may help reduce unnecessary diagnostic surgery. However, their routine use may be limited by cost, accessibility, and variability across healthcare settings. Therefore, readily available and cost-effective biomarkers, such as thyroid hormone sensitivity indices, may serve as complementary tools to established diagnostic strategies (18–22).

The Bethesda System is widely used in thyroid cytopathology; however, the management of nodules classified as Bethesda category III remains challenging (18). Repeat fine-needle aspiration biopsy (FNAB) is commonly used to refine diagnosis, although indeterminate results may persist in a subset of cases (19). In our study, 89.2% of patients initially diagnosed with AUS underwent repeat FNAB, resulting in reclassification to Bethesda II in 51.4% of cases, while 21.6% remained Bethesda III. These findings highlight the role of repeat FNAB in reducing diagnostic uncertainty and guiding clinical management. Following the initial AUS diagnosis, 40% of patients ultimately underwent surgery, whereas 42.7% were managed with continued ultrasonographic surveillance. The overall malignancy rate among surgically treated patients was 30.8%, which is consistent with previously reported rates ranging from approximately 26% to 37.8% in Bethesda category III nodules (17, 23).

Several studies have shown that suspicious ultrasonographic features such as hypoechogenicity, irregular margins, microcalcifications, and increased vascularity are predictive factors for malignancy in Bethesda category III nodules (24). In our study, hypoechogenicity, the presence of microcalcifications, irregular margins, and intranodular vascularity were identified as significant ultrasonographic predictors of malignancy. Gao et al. reported that the presence of any one of the suspicious ultrasonographic features—such as hypoechogenicity, calcifications, irregular margins, or a taller-than-wide shape—had a pooled sensitivity of 0.75 and specificity of 0.48 in predicting malignancy (25). Similarly, Alshahrani et al. identified hypoechogenicity, microcalcifications, and irregular margins as suspicious features associated with malignancy in Bethesda category III nodules, consistent with the findings of our study (26). Some studies in the literature have evaluated diagnostic performance of various ultrasound risk stratification systems for indeterminate nodules, specifically Bethesda categories III and IV. Studies using ACR TI-RADS found it to be ineffective in predicting malignancy in Bethesda category III nodules (27, 28). In a study by Słowińska-Klencka and et all involving 127 AUS nodules, EU-TIRADS showed higher accuracy for AUS nodules. In addition to individual ultrasonographic features, the EU-TIRADS classification emerged as the strongest predictor of malignancy in the multivariable analysis, highlighting the importance of structured ultrasound risk stratification systems in the evaluation of indeterminate thyroid nodules. The nodules classified into category 5 of EU-TIRADS had a strongly increased risk of malignancy (17). In our study demonstrated malignancy risk rates of 0% in TR2, 10.5% in TR3, 28.1% in TR4, and 61.4% in TR5 categories, indicating that the EU-TIRADS classification was effective in differentiating benign from malignant nodules.

A family history of thyroid cancer has been recognized as a potential clinical risk factor for thyroid malignancy. Population-based studies have demonstrated that first-degree relatives of patients with thyroid cancer have a three- to five-fold higher risk of developing the disease compared with individuals without such a history (29). Our findings support the potential role of family history as a clinical risk factor that should be considered during the evaluation of AUS nodules.

Systemic inflammatory responses have been shown to influence tumor growth and metastasis. Hematological indices derived from peripheral blood counts, such as platelet count, mean platelet volume (MPV), neutrophil-to-lymphocyte ratio (NLR), and platelet-to-lymphocyte ratio (PLR), have been associated with clinical and pathological features as well as survival outcomes in various malignancies. However, the diagnostic value of these indices in thyroid cancer remains controversial. For example, Machairas et al. reported that these hematological indices were not helpful in differentiating benign goiter from thyroid cancer (30). Similarly, previous studies have reported inconsistent findings regarding PDW levels in papillary thyroid carcinoma, with some studies showing lower and others higher PDW values compared with benign thyroid conditions (11, 31, 32). While several studies have suggested that PLR and NLR may help distinguish malignant from benign thyroid nodules, other reports have not confirmed this association (33, 34). Consistent with these findings, PLR and NLR were not significantly associated with malignancy in our cohort. Bostan et al., in a study involving 200 patients with AUS nodules, also reported that NLR has limited value as a biomarker for predicting malignancy, although values ≥2.24 may indicate an increased malignancy risk requiring closer follow-up (35). Platelet-related indices have also been investigated as potential inflammatory markers in thyroid cancer. Previous studies have suggested that the PLR-to-PDW ratio may help distinguish papillary thyroid carcinoma from benign thyroid nodules (14). In our study, although the PLR-to-PDW ratio remained significantly associated with malignancy in multivariable logistic regression analysis, it demonstrated limited diagnostic performance in ROC analysis. An additional methodological consideration is the potential time interval between fine-needle aspiration biopsy (FNAB) and blood sampling. Although laboratory parameters were recorded at the time of AUS diagnosis, variability in the timing of blood collection in routine clinical practice may have introduced heterogeneity, particularly in inflammatory indices, which are more susceptible to short-term fluctuations. However, in our study, inflammatory markers such as NLR and PLR did not demonstrate significant associations with malignancy, suggesting that any potential timing-related variability did not materially influence the primary findings. In contrast, thyroid hormone sensitivity indices are relatively stable and less likely to be affected by short-term temporal variations, supporting the robustness of the observed associations.

Thyroid-stimulating hormone (TSH), secreted by the anterior pituitary gland, plays a central role in the regulation of thyroid function and thyroid cell proliferation. Previous studies have demonstrated a positive association between TSH levels and thyroid cancer risk (36). In our study, a TSH cutoff value of ≥1.83 was associated with an increased risk of malignancy (OR: 2.59), with a sensitivity of 49% and specificity of 71%. Although some studies have suggested that elevated anti-thyroid peroxidase (anti-TPO) and anti-thyroglobulin (anti-Tg) antibody levels may be associated with papillary thyroid carcinoma (9), these antibodies did not show a significant predictive value for malignancy in our cohort. Beyond TSH levels alone, increasing attention has been directed toward indices reflecting thyroid hormone sensitivity. Alterations in thyroid hormone sensitivity may disrupt the feedback regulation of the hypothalamic–pituitary–thyroid axis and potentially contribute to tumorigenesis. Several indices have been developed to evaluate central thyroid hormone sensitivity, including the thyrotropic thyroxine resistance index (TT4RI), thyroid-stimulating hormone index (TSHI), and thyroid feedback quantile-based index (TFQI). Previous studies have demonstrated that impaired central thyroid hormone sensitivity is associated with metabolic disorders such as metabolic syndrome, diabetes, hyperuricemia, cardiovascular disease, and increased visceral fat area (16). To our knowledge, our study is the first to evaluate the relationship between thyroid hormone sensitivity indices and malignancy risk in patients with thyroid nodules initially classified as Bethesda category III. In our analysis, TT4RI ≥27.67 predicted malignancy with a sensitivity of 49.1% and specificity of 75%, whereas TSHI ≥2.6 showed a sensitivity of 50.8% and specificity of 74.2%. Taken together, these findings indicate that thyroid hormone sensitivity indices do not overcome the limited sensitivity of standalone biochemical risk stratification in AUS nodules. However, their ability to increase specificity when used in conjunction with suspicious ultrasonographic features suggests that these indices may be more useful as complementary diagnostic markers than as screening tools. Therefore, in clinical practice, the most appropriate role of these indices may be as adjunctive biochemical parameters that provide additional value to ultrasonographic risk assessment. Future studies are needed to evaluate whether these indices provide incremental diagnostic value across different ultrasonographic risk categories, particularly in nodules with low or intermediate EU-TIRADS classification. These findings suggest that decreased central sensitivity to thyroid hormones may be associated with malignant transformation in thyroid nodules. One possible explanation is that reduced sensitivity of the hypothalamic–pituitary–thyroid axis leads to relatively elevated TSH levels, which may stimulate thyroid cell proliferation. TSH signaling has been shown to promote vascular endothelial growth factor (VEGF) secretion, thereby inducing neoangiogenesis and potentially accelerating genomic instability in thyroid cancer (37). In addition, TSH signaling may influence tumorigenesis through the modulation of p53 protein expression (38). We hypothesize that inflammation associated with malignancy may contribute to impaired central thyroid hormone sensitivity through reduced activity of type 2 deiodinase (DIO2) in the pituitary gland, leading to increased circulating TSH levels. However, further studies are required to clarify the underlying mechanisms.

A limitation of the present study is that no formal *a priori* sample size calculation was performed due to its retrospective cross-sectional design. Instead, all consecutive patients who met the predefined inclusion and exclusion criteria during the study period were included to minimize selection bias and to reflect routine clinical practice. The available sample size may have limited statistical precision, particularly in subgroup analyses and for associations characterized by relatively wide confidence intervals. An additional important limitation is the potential for selection bias and partial verification bias due to incomplete histopathologic confirmation. Patients selected for surgery were likely to have a higher baseline suspicion of malignancy based on cytologic, ultrasonographic, and clinical features, which may have influenced both the observed malignancy rate and the estimated predictive performance of the evaluated indices. This approach is consistent with current guideline-based clinical management of AUS nodules. Therefore, our findings should be interpreted as reflecting predictors of malignancy within a clinically managed AUS cohort rather than definitive diagnostic accuracy estimates applicable to all AUS nodules. This study was conducted in a single tertiary care center, which may limit the generalizability of our findings to broader populations or different clinical settings. Furthermore, the retrospective design introduces inherent limitations, including the potential for unmeasured confounding factors and incomplete or missing data. Prospective, multicenter studies are needed to validate our findings and further clarify the clinical utility of thyroid hormone sensitivity indices.

In conclusion, thyroid hormone sensitivity indices, particularly TSHI and TT4RI, may provide additional information for malignancy risk stratification in thyroid nodules initially classified as Bethesda category III. When used alongside ultrasonographic risk stratification systems, these indices may help improve diagnostic evaluation and follow-up strategies in patients with AUS nodules who are not referred for immediate surgery.

## Data Availability

The original contributions presented in the study are included in the article/supplementary material. Further inquiries can be directed to the corresponding author.
